# Phytoradiotherapy to enhance cancer treatment outcomes with cannabidiol, bitter melon juice, and plant hemoglobin

**DOI:** 10.3389/fonc.2022.1085686

**Published:** 2023-01-26

**Authors:** Tyler Alfonzetti, Michele Moreau, Sayeda Yasmin-Karim, Wilfred Ngwa, Stephen Avery, Denisa Goia

**Affiliations:** ^1^ Department of Radiation Oncology, Perelman Center for Advanced Medicine, University of Pennsylvania, Philadelphia, PA, United States; ^2^ Department of Radiation Oncology and Molecular Radiation Sciences, Johns Hopkins University, Boston, MA, United States; ^3^ Department of Radiation Oncology, Brigham and Women’s Hospital and Harvard Medical School, Boston, MA, United States; ^4^ Department of Radiation Oncology, Dana Farber Cancer Institute and Harvard Medical School, Boston, MA, United States

**Keywords:** phytoradiotherapy, radiotherapy, phytomedicines, cannabidiol (CBD), bitter melon juice (BMJ), pancreatic adenocarcinoma, plant hemoglobin, anemia

## Abstract

Despite technological advances in radiation therapy for cancer treatment, many patient populations still experience mediocre survival percentages, local control, and quality of life. Additionally, much of the world lacks access to expensive, modern treatment options. The need for innovative, cost-effective solutions that can improve patient treatment outcomes is essential. Phytomedicines have been shown to induce apoptotic tumor cell death, diminish tumor progression, reduce cancer incidence, alleviate harmful hypoxic conditions, and more. While an ample amount of research is available that characterizes many phytomedicines as having anti-cancer properties that increase tumor cell killing/control and mitigate the harmful side effects of radiation damage, little work has been done to investigate the synergistic effect of phytoradiotherapy: combining radiation treatment with phytomedicines. In this study, a protocol for testing the radiosensitizing effects of phytomedicines was validated and used to investigate the well-known plant based medicine cannabidiol (CBD) and the lesser-known medicinal fruit Bitter Melon. Additionally, based on its high concentration of plant hemoglobin which has been shown to abate hypoxia, the African-indigenous *Justicia* plant was tested in pancreatic adenocarcinoma mouse models. The studies reveal that these phytomedicines can effectively enhance tumor cell killing, minimize tumor growth, and prolong mice survival. There is certainly the need for additional research in this regard, however, phytoradiotherapy: the use of phytomedicines to enhance radiation therapy treatment outcomes, continues to show potential as a promising, innovative way to improve cancer care.

## 1 Introduction

Phyto-, or plant based, medicines have been extensively researched throughout human history demonstrating a remarkable variety of treatment applications (1–3). Specifically, a number of phytomedicines have displayed anti-tumorigenic properties for a breadth of cancer types including leukemia, pancreatic, lung, abdominal, brain, cervical, and breast (4). The wide scope of phytomedicines available and their natural ability to counter tumor progression is one of the reasons why the term “Phytoradiotherapy” has been coined in previous research (4). Meaning “radiation therapy delivered concurrently with phytomedicines”, Phytoradiotherapy presents a unique way to enhance radiation therapy for the treatment of cancer while mitigating damaging side effects. Their antioxidant properties (5), reduction of hypoxia (6), and historical efficacy to subdue fatigue, nausea, skin irritation, and other radiation induced side-effects make it possible for phytomedicines to act as a double-edged sword when used in conjunction with radiotherapy.

Besides the therapeutic benefits, the growing economic burden of cancer treatment (7, 8), lack of access to expensive synthetic drugs for low/middle income countries, and global recognition of plant based medicine applications (9, 10), are also worth considering. Specifically, the economic impact of phytoradiotherapy would be three-fold: reducing the cost of cancer treatment universally, providing affordable care for patients in developing countries, and providing economic opportunity (4).

For these reasons and more, researchers at Harvard investigated the use of cannabinoids (CBD) as the first phytomedicine for Phytoradiotherapy (11). Chosen for its extensively acknowledged medicinal properties (12–14), it was used in conjunction with radiotherapy to treat human pancreatic cancer cells *in vitro*, and human lung cancer in mice. The results of the study showed that CBD treatment substantially enhanced tumor cell killing, prevented tumor-progression, and prolonged mice survival (11), but called for additional research in this area to be conducted due to its novelty and the lack of related research. For this reason, research presented in this article was conducted to verify the original results of the Harvard study (11). Once verified, the same protocol was used to test another phytomedicine which has previously shown to be effective against pancreatic cancer: Bitter Melon.

The Bitter Melon indigenous to Asia, Africa, and the Caribbean is another phytomedicine with documented anti-cancer properties. Specifically, one study experimented with pancreatic tumors in nude mice. The results of their study showed that being treated with Bitter Melon Juice (BMJ) induced strong apoptotic cell death, decreasing cell viability in all four of the different pancreatic cancer cell lines examined. The same study also provided evidence that oral administration of BMJ over the course of 6 weeks inhibited tumor growth in nude mice (15). With pancreatic being among the deadliest types of cancer with just a roughly 9% 5-year survival rate (16), concurrent radiotherapy and phytomedicine treatment demonstrates an exciting initial promise. In a 2017 study, Bitter Melon Extract’s (BME) anti-tumor properties were tested in mouse models. It was found that the extract exhibited strong anti-proliferative effects by inducing caspase-dependent apoptosis in breast cancer cells. In the same study, BME enhanced autophagy, recently deemed an important method of tumor killing, and similar to its effect against pancreatic cancer, inhibited tumor growth in mice (17). Here, we investigate the potential to enhance radiotherapy treatment outcomes with concurrent BMJ treatment.

One mechanism by which phytomedicines induce a radiosensitizing effect is their ability to mitigate hypoxic conditions within a tumor microenvironment (4). Intratumoral hypoxia is a major regulator of radio-resistance in cancer treatment due to the reduction in blood supply from immature blood vessels and the necrotic tumor core (18). Anemia, a disease affecting over 2 billion people around the globe (18), is often a symptom of cancer that increases the degree of hypoxia (4). This poses a problem for patients that rely on radiation therapy for curative treatment and calls for a solution that mitigates intratumoral hypoxia. One phytomedicine, the African-indigenous *Justicia* plant (Acanthaceae family), has been revolutionized to contain “plant hemoglobin” which is similar to human α and β hemoglobin. This is in contrast to *Justicia* plant found in other areas where it typically has rudimentary hemoglobin called hemoglobin ɣ (18). Work by Woods et al. showed that the extraordinarily high hemoglobin concentrations in the isolated leaf extracts contained essential components for human blood health. It also revealed that cancer cells (PC3, human prostate cancer cells and A549, adenocarcinoma human alveolar basal epithelial cells) showed reduced survival when treated with the purified plant extract while normal cells (HUVECS, human umbilical vein endothelial cells and RWPE, normal prostate epithelial cells) experienced little to no effect (18). Additionally, female mice with chronic hemorrhagic anemia treated with *Justicia* extract showed a more rapid increase in hemoglobin level than the control group treated with saline (18). These effects indicate the need for further research while showing promise that African-indigenous *Justicia* is a natural medicine that can potentially treat Anemia and be an effective phytoradiotherapy agent. Here, we investigate the ability for this plant extract (Plant Hemoglobin, Plant HB) to enhance tumor control by increasing the radiosensitivity of tumor cells.

## 2 Material and methods

### 2.1 Cell culture and materials

Human, Epithelial pancreatic adenocarcinoma cell line PANC02 (ATCC) was maintained in DMEM media supplemented with 10% Fetal Bovine Serum (FBS) and 1% antibiotics (penicillin) following standard protocol. Cells were maintained at 37°C in a humidified incubator under 5% CO_2_ atmosphere according to standard protocol. All supplements were obtained from Sigma-Aldrich and tissue culture plastics were obtained from Corning Life Sciences. All experimental cells were at least 95% alive.

### 2.2 Clongenic cell survival assay

PANC02 cells from an actively growing monolayer were trypsinized and 300 cells per well were seeded in 6-well plates. After incubating for 24 hours, seeded cells were treated with 0, 1, 2, and 5 µg/well of CBD concentrations. Following another 24 hours of incubation, the well plates were irradiated at: 0, 2, 4 and 6 Gy using 320kVp energy, 12.50mA and the following filters: 1.5mm Al, 0.25mm Cu, 0.75mm Sn. Precision’s X-RAD 320ix was used for irradiation. The growing colonies (>50 cells/colony) were fixed with 75% ethanol and stained with 1% crystal violet one week after irradiation. Colonies were counted using imageJ software and a percent survival curve was generated according to standard procedure (19).

The above procedure was repeated with identical concentrations of Bitter Melon Extract dissolved in DMEM medium rather than CBD.

### 2.3 *In-Vivo* studies with plant hemoglobin

Lyophilized *Justicia* plant extract was analyzed for its hemoglobin content (18). ELISA was performed using the human hemoglobin kit (Sigma-Aldrich) to quantify the hemoglobin concentration (human type) (18). A western blot assay (using human hemoglobin α and β antibody) was also used to confirm the presence of α and β hemoglobin chains in the plant hemoglobin (18). A subcutaneous pancreatic tumor mouse model was generated in wild C57BL/6NTac mice from Taconic Bioscience by implanting a syngeneic pancreatic adenocarcinoma cell line, PANC02 (National Cancer Institute). One subcutaneous tumor was generated on one flank of each mouse by injecting 1.5x10 (5) cells/tumor suspended in PBS (11). Only cells with > 90% viability were implanted. Two weeks after the implant, mice with generated tumors were randomized and treated with filtered (0.2µm filter, BD Bioscience) Plant Hb (0.1mg/kg dissolved in 100 µl of PBS, one time for each mouse tumor) intratumorally just before the radiation treatment. For the control group of mice, only PBS (100 µl) was used.

A single 10 Gy fraction of radiation treatment was given within 15 minutes after the Plant Hb injection. A Small Animal Radiation and Research Platform (SARRP, Xstrahl, Inc, Suwanee, GA) was used for image guided-radiation treatment (IGRT) only to the tumor under CT image guidance at 220 kVp, 13 mA, and a 0.15 mm copper filter with. Tumor measurement (length and width) and survival analysis were performed following standard protocol (11). Volume of the tumors was analyzed following the formula: tumor volume = [1/2 * L * W (2)] where L and W are the length and widths of the tumor, respectively. The survival percent and statistical analysis were performed using GraphPad Prism V9. The mice were maintained, and the study was conducted, at Dana Farber Cancer Institute (DFCI) Animal Facility under DFCI IACUC-approved protocol (DFCI IACUC-15-040).

## 3 Results

### 3.1 Validation of CBD induced radiosensitization

**Figure 1:**
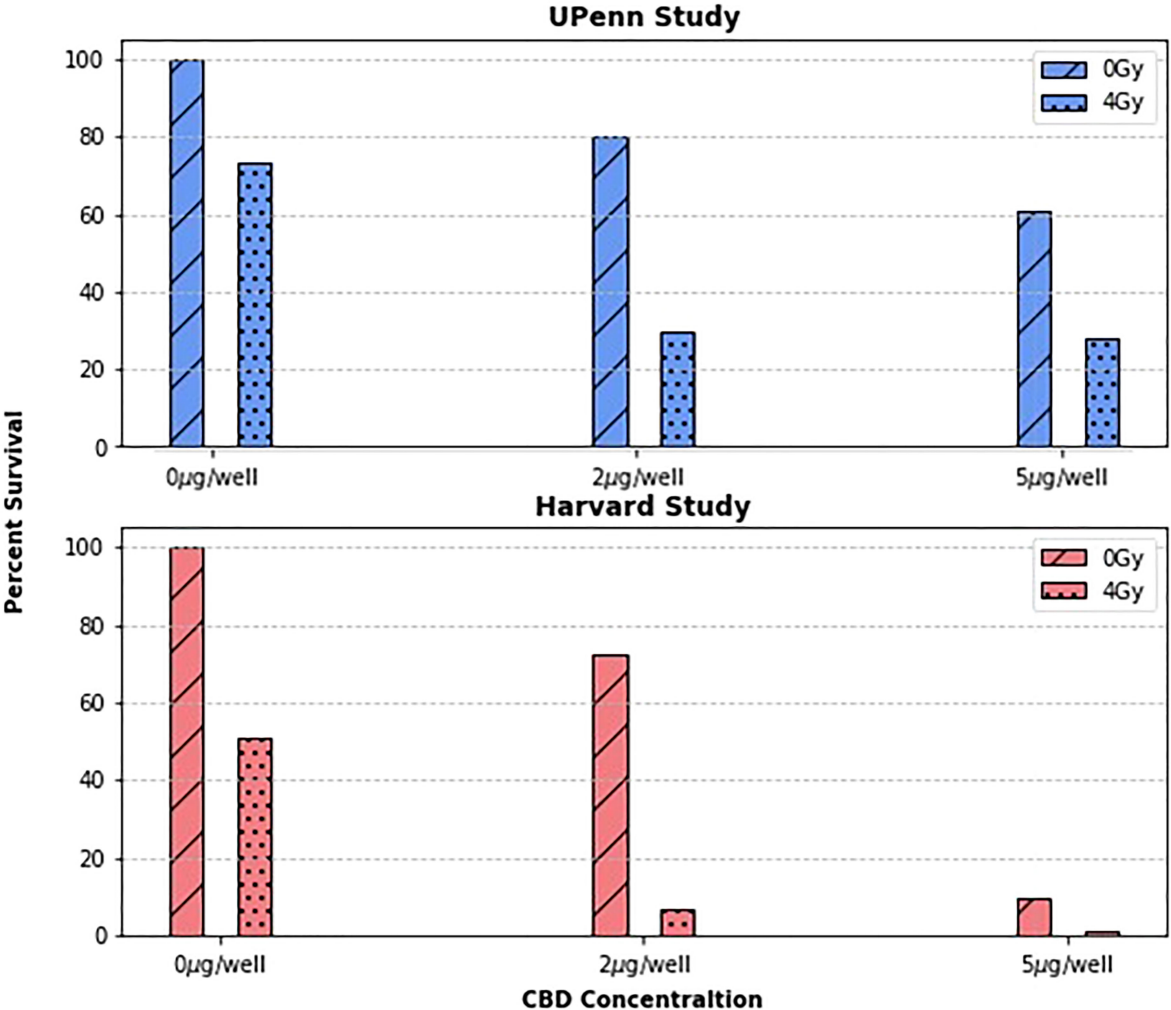
The surviving fraction at concentrations of 0, 2, and 5µg/well and dose levels of 0 and 4Gy were compared between two research groups. As expected, the surviving fractions vary quantitatively between groups, but both group’s data suggests a clear trend: that treating cancer cells with CBD enhances their radiosensitivity.

To first validate the previous work done by Yasmin-Karim et al (11) which showed that CBD treatment enhances tumor cell killing *in vitro*, the original Harvard study results were reproduced in **Figure 1** and compared to those from the new UPenn Study. Due to slightly different irradiation settings, and the general variability of biological experiments, it is difficult to directly compare the two studies quantitatively. What is more important, however, is that the two plots tell the same story qualitatively. The UPenn study confirms the effect of concurrent radiotherapy and CBD treatment on PANC02 cells showing that as the concentration of CBD increases, so does tumor cell killing. This agreement between the independently measured clonogenic assays builds confidence in the radiation enhancing effects of CBD and also demonstrates the reproducibility of the protocol developed to test the radiotherapy enhancing effects of phytomedicines *in vitro*.

### 3.2 CBD and BMJ cell survival curves

**Figure 2:**
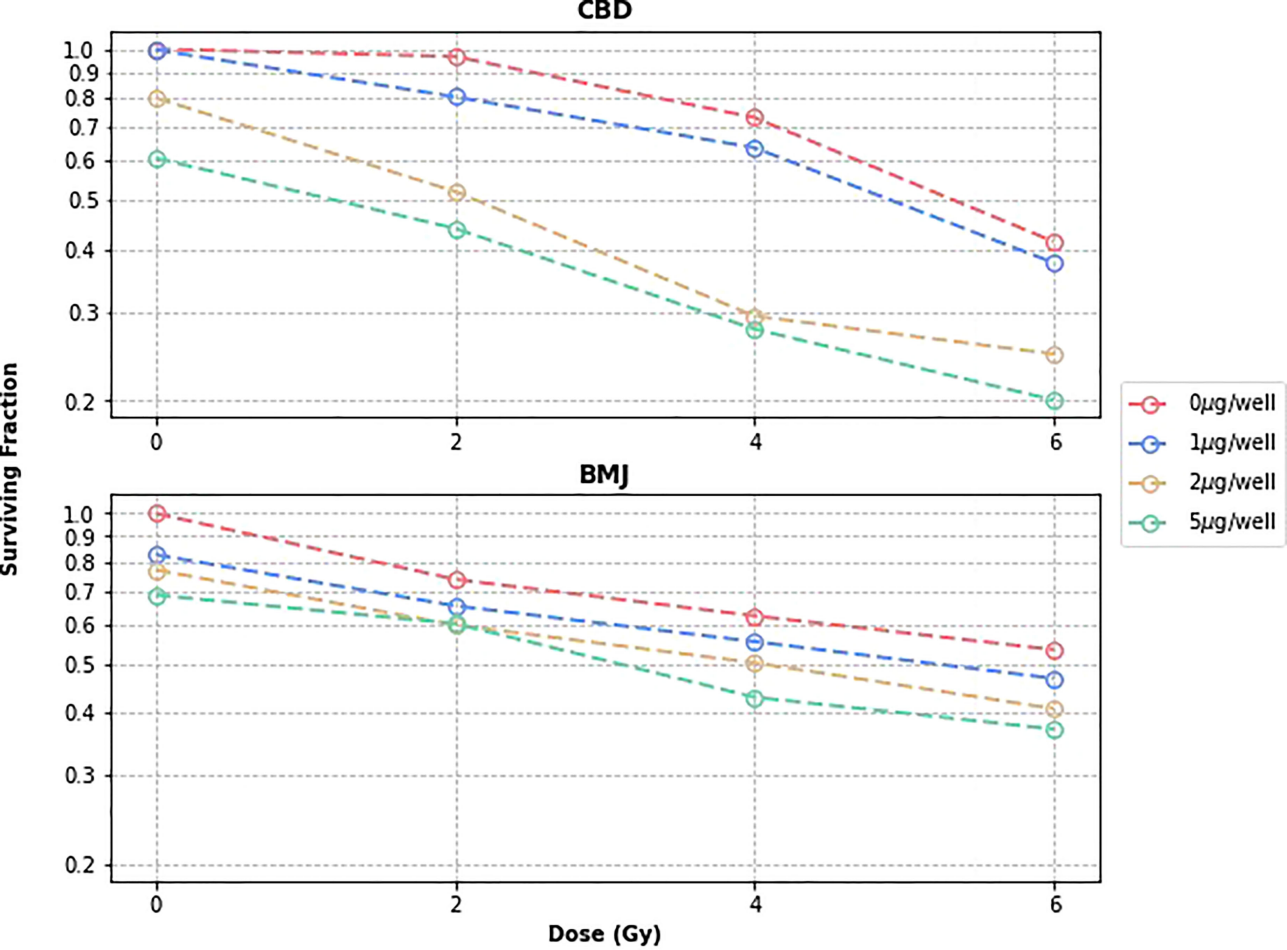
CBD and BMJ Cell Survival Curves.

In addition to the dose levels shown in **Figure 1**, 2Gy and 6Gy dose levels were investigated as well. Furthermore, a CBD concentration of 1μg/well was investigated in addition to 0μg/well, 2μg/well, and 5μg/well previously tested. Identical dose levels and phytomedicine concentrations were also used to investigate the radiotherapy enhancing effects of BMJ. The cell survival curves for both phytomedicines are shown in **Figure 2**.

#### 3.2.1 CBD

One feature of the CBD clonogenic survival curve of interest is the slight “shoulder” evident for cells treated without CBD and with 1μg/well. A shoulder on a tumor cell survival curve with the dose as the independent variable is known to be associated with the ability of tumor cells to repair non-lethal damage at lower doses (20). The linear quadratic model, a popular choice to roughly describe the behavior of cell survival curves, explains this as the combined effect of cell death from single lethal “hits” and cell death occurring from multiple lethal “hits” as described in literature (21, 22). At lower doses, the single hit linear term dominates and is responsible for the “shouldering” of the curve due to the fact that single hit events are easier for cells to repair than multiple hits. On the same figure, curves corresponding to cells treated with higher concentrations of CBD/well do not show a shoulder at all. It is possible then that the reason why no shoulder is seen on curves corresponding to cells treated with higher concentrations of CBD (2μg/well, 5μg/well) is due to the phytomedicine preventing effective single-hit cellular repair. This would further explain the more pronounced drop in cell survival from curves treated with 0μg/well to those treated with 2μg/well compared to the drop from curves treated with 2μg/well to 5μg/well. This of course cannot be definitively proven without conducting a biological study investigating the effect of CBD on tumor cell repair mechanics. Although studies exist that investigate the detailed biological mechanisms that CBD induces in a variety of cancer types (23), similar studies involving ionizing radiation are lacking.

#### 3.2.2 BMJ

From the BMJ cell survival curve in **Figure 2** it is clear to see the overall trend is similar to that of cells treated with CBD; As cells are treated with greater concentrations of BMJ, their surviving fraction decreases. Although the overall trend is the same, the shape of BMJ and CBD survival curves are not identical. At lower concentrations of phytomedicine, where a shoulder region was seen for CBD survival curves, there is no obvious shoulder region in the BMJ survival curves. This is not expected, since the 0μg/well curves (black) should be very similar since they represent identical scenarios: PANC02 cells being irradiated to different levels of dose. However, the fact that cells were not grown, treated, irradiated, and counted at the exact same time could explain the difference in shape (24). Generally speaking the survival curves are more linearly shaped for cells treated with bitter melon than those treated with CBD. At the 2Gy dose level, data showed that a BMJ treatment of 2μg/well did more cell killing than a BMJ treatment of 5μg/well. As this discrepancy is only slight (the difference in surviving fraction is less than 1%) and all other data follows the same trend, it is inferred that this is an outlier data point. Despite any minor differences in shape, the survival curve for cells treated with BMJ tells a similar story those for cells treated with CBD: bitter melon is an effectively radiosensitizing phytomedicine with potential to enhance radiation therapy treatment outcomes.

#### 3.2.3 CBD vs. BMJ comparison

**Figure 3:**
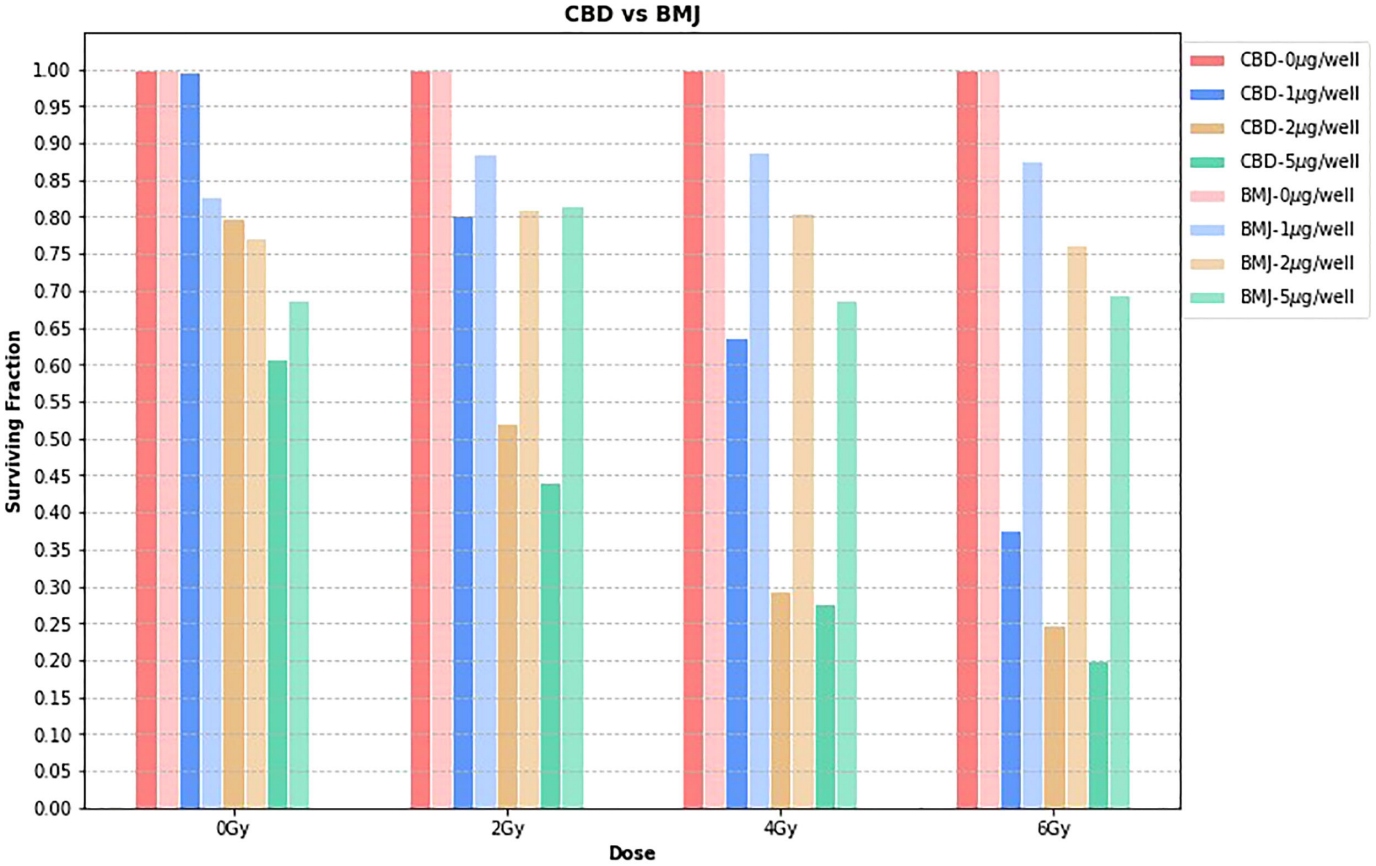
CBD vs. BMJ. At each dose level, the surviving fraction is normalized to the 0μg/well value for that particular dose level. This is a way to isolate the effects of the phytomedicine treatment from the effect of the ionizing radiation.

**Figure 3** shows the surviving fraction of CBD vs BMJ at each dose level, for each concentration. The surviving fractions for each phytomedicine concentration are normalized to the 0μg/well value. Since the time of growth, incubation, phytomedicine treatment, and irradiation were not simultaneous in this study, quantitative comparison between CBD and BMJ can be slightly misleading. Here, we focus on a general, qualitative comparison of the radiosensitizing capabilities of both phytomedicines. The obvious trend from **Figure 3** is that, compared to CBD, BMJ of the same concentration provided less cell killing (as seen by taller bars which indicate a higher surviving fraction). The difference is less pronounced at lower radiation doses, but becomes increasingly more noticeable at higher doses. This could indicate the enhancement of ionizing radiation damage of CBD is more potent than that of BMJ. It is still apparent from **Figure 3**, however, that increasing concentrations of BMJ results in a greater proportion of cell death.

### 3.3 Radiation enhancing effect of plant hemoglobin

**Figure 4:**
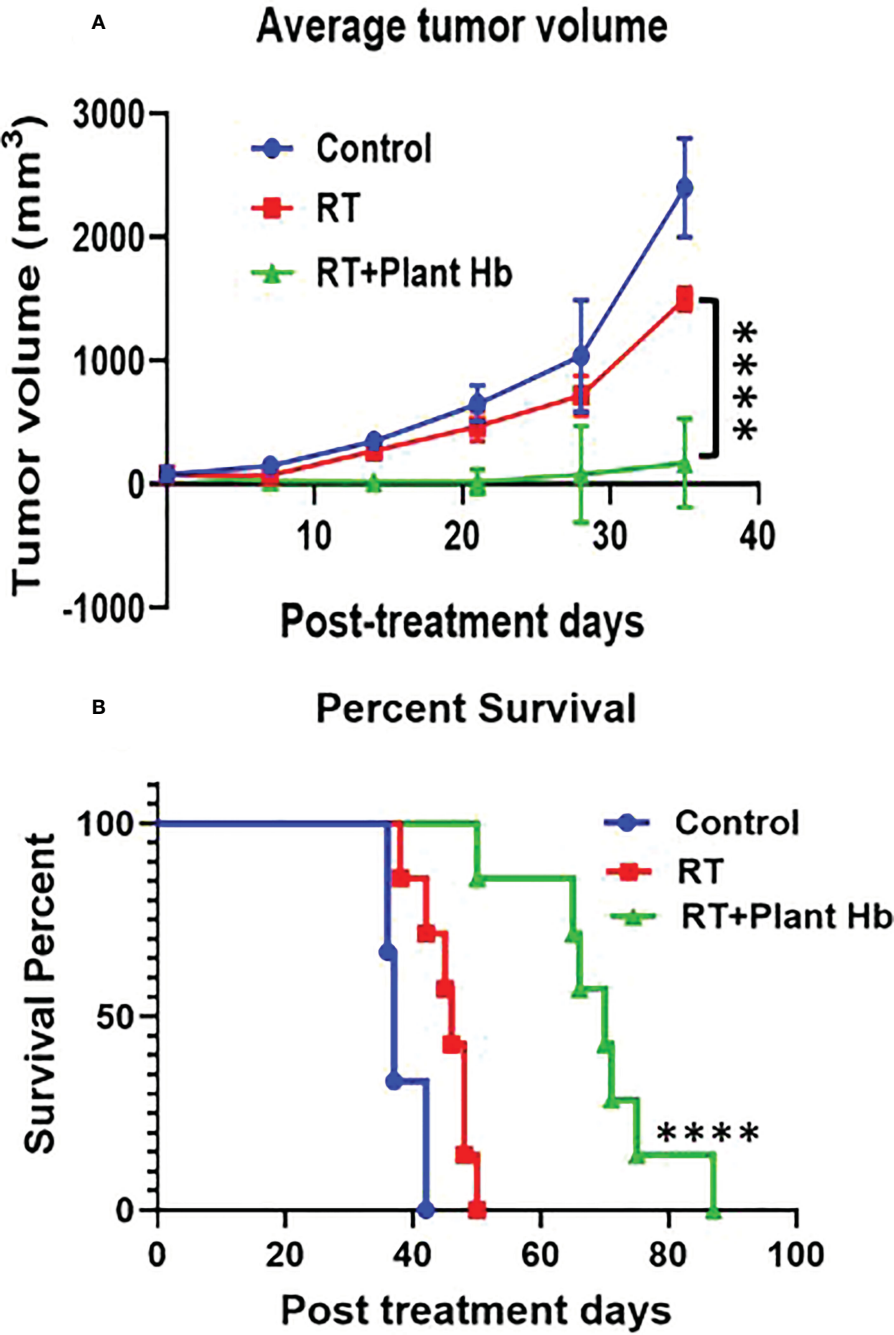
Plant Hemoglobin as a Radiation Therapy Enhancer. Significant reduction of tumor volume (n=6) **(A)** and increased survival (n=7) **(B)** in mice with pancreatic adenocarcinoma after treatment with plant hemoglobin and adjuvant radiation therapy (****p<0.0001).

**Figure 4** shows the tumor volume and survival percentage of mice with subcutaneous pancreatic adenocarcinoma as a function of post treatment days for three different treatments: control, radiation therapy alone, and phytoradiotherapy with plant hemoglobin as the phytomedicine agent. For the phytoradiotherapy group, well-characterized plant hemoglobin was injected intratumorally immediately before radiation treatment to 10Gy in the pancreatic adenocarcinoma mouse model. The control and radiation groups were treated with same amount of PBS injected intratumorally, and the radiation alone group was additionally treated with a single 10Gy fraction. The radiosensitizing properties of the plant extract are clear with significant tumor volume reduction (p<0.0001) observed in the phytoradiotherapy group compared to the cohort with only radiation treatment (**Figure 4A**). Significant increase in percent survival and duration of survival (p<0.0001) were also observed in this combination-treated group (**Figure 4B**) confirming the significant radio-enhancing effect of plant hemoglobin for cancer treatment.

## 4 Discussion

Results showing that both CBD and BMJ are effective radiosensitizing phytomedicines demonstrate promise that the two plant-based medicines have a potential future in radiation therapy as treatment enhancing drugs at a much more affordable rate than their synthetic alternatives. The advancement of highly customizable drug delivery techniques generates further promise for phytoradiotherapy (11). Human clinical trials are an important step in integrating such phytomedicines into the clinic, however there is plenty of relevant research that can be conducted *in vitro* and in animal studies. For example, CBD and BMJ represent only two out of the vast collection of available and promising “anticancer” phytomedicines. The protocol, developed by Yasmin-Karim et al (11, 25, 26), and validated here, can serve as a standardized way of preliminary testing/comparing different phytomedicines as radiosensitizers. Additionally, the hypothesis that phytomedicines can both enhance tumor cell killing while offering protective properties for normal tissue calls for additional research to be conducted. Specifically, this protocol should be tested on normal, in addition to tumorigenic cells to investigate the relative effect of phytoradiotherapy on healthy versus cancerous tissue. If phytomedicine treatment enhances tumor cell killing to a much higher degree than healthy cell killing (or decreases healthy cell killing), additional support for Phytoradiotherapy is established.

Comparing the ability of phytomedicines to alter ionizing radiation treatment outcomes of healthy cells could reveal important information about the ability of different phytomedicines to preferentially combat tumor cells while sparing normal tissue. A specific application of interest is the ability to mitigate hypoxic conditions often present within a tumor microenvironment. Significant hypoxia exists to some degree in most solid tumors due to inadequate oxygen delivery of the abnormal vasculature which cannot meet the demands of the rapidly proliferating cancer cells. Intratumoral hypoxia is a master regulator of radioresistance and immune suppression. Because, in radiotherapy, the primary mechanism of cell killing is the creation of reactive oxygen species, hypoxic tumors are often radiation-resistant with poor survival outcomes. The strategy of phytoradiotherapy with plant hemoglobin can increase the radiation effect in solid tumors to develop an innovative way to enhance radiotherapy treatment outcomes. The results of this study would be enhanced with an investigation into the radiosensitization ability of Plant Hb *via* the *in-vitro* protocol described in this manuscript and with investigation into hypoxia levels in the mouse models prior, during, and after treatment. The need for additional research pertaining to the combination of radiation therapy and plant based medicines is emphasized in this article.

## Data availability statement

The raw data supporting the conclusions of this article will be made available by the authors, without undue reservation.

## Ethics statement

The animal study was reviewed and approved by Institutional Animal Care and Use Committee (IACUC) of the Dana-Farber Cancer Institute (DFCI).

## Author contributions

TA is a Medical Physics Resident and first author. WN and SA are research mentors and collaborators. SY-K and MM are collaborators that have contributed data towards the use of phytomedicines. DG is a collaborator who aided in the BMJ and CBD clonogenic assay studies at the University of Pennsylvania. All authors contributed to the article and approved the submitted version.

## References

[1] Ahuja A Gupta J Gupta R. Miracles of herbal phytomedicines in treatment of skin disorders: Natural healthcare perspective. Infect Disord - Drug Targets (2021) 21(3):328–38. doi: 10.2174/1871526520666200622142710 32568024

[2] Hudson JB. Applications of the phytomedicine echinacea purpurea (purple coneflower) in infectious diseases. J BioMed Biotechnol (2012) 2012:1–16. doi: 10.1155/2012/769896 22131823 PMC3205674

[3] Iyer M Gujjari A Rao R Gowda D Srivastava A. Biomedical applications of phytomedicines: Dental perspective. Dent Hypotheses (2016) 7(2):34. doi: 10.4103/2155-8213.183757

[4] Alfonzetti T Yasmin-Karim S Ngwa W Avery S. Phytoradiotherapy: An integrative approach to cancer treatment by combining radiotherapy with phytomedicines. Front Oncol (2021) 10:624663. doi: 10.3389/fonc.2020.624663 33628736 PMC7898963

[5] Kahl R Kappus H. Toxikologie der synthetischen antioxidantien bha und bht im vergleich mit dem natürlichen antioxidans vitamin e. Z für leb und -forsch (1993) 196(4):329–38. doi: 10.1007/bf01197931 8493816

[6] Semenza GL. Hypoxia-inducible factors: Mediators of cancer progression and targets for cancer therapy. Trends Pharmacol Sci (2012) 33(4):207–14. doi: 10.1016/j.tips.2012.01.005 PMC3437546 22398146

[7] Rahman F Seung SJ Cheng SY Saherawala H Earle CC Mittmann N. Radiation costing methods: A systematic review. Curr Oncol (2016) 23(4):392–408. doi: 10.3747/co.23.3073 PMC4974046 27536189

[8] Siddiqui M Rajkumar SV. The high cost of cancer drugs and what we can do about it. Mayo Clin Proc (2012) 87(10):935–43. doi: 10.1016/j.mayocp.2012.07.007 PMC3538397 23036669

[9] Rashrash M Schommer JC Brown LM. Prevalence and predictors of herbal medicine use among adults in the united states. J Patient Exp (2017) 4(3):108–13. doi: 10.1177/2374373517706612 PMC5593261 28959715

[10] Moreau M Ibeh U Decosmo K Bih N Yasmin-Karim S Toyang N. Flavonoid derivative of cannabis demonstrates therapeutic potential in preclinical models of metastatic pancreatic cancer. Front Oncol (2019) 9:660. doi: 10.3389/fonc.2019.00660 31396485 PMC6663976

[11] Yasmin-Karim S Moreau M Mueller R Sinha N Dabney R Herman A. Enhancing the therapeutic efficacy of cancer treatment with cannabinoids. Front Oncol (2018) 8:114. doi: 10.3389/fonc.2018.00114 29740535 PMC5928848

[12] Velasco G Sánchez C Guzmán M. Anticancer mechanisms of cannabinoids. Curr Oncol (2016) 23(11):23–32. doi: 10.3747/co.23.3080 PMC4791144 27022311

[13] Massi P Vaccani A Ceruti S Colombo A Abbracchio MP Parolaro D. Antitumor effects of cannabidiol, a nonpsychoactive cannabinoid, on human glioma cell lines. J Pharmacol Exp Ther (2004) 308(3):838–45. doi: 10.1124/jpet.103.061002 14617682

[14] Dariš B Tancer Verboten M Knez Ž Ferk P. Cannabinoids in cancer treatment: Therapeutic potential and legislation. Bosn J Basic Med Sci (2019) 19(1):14–23. doi: 10.17305/bjbms.2018.3532 30172249 PMC6387667

[15] Kaur M Deep G Jain AK Raina K Agarwal C Wempe MF. Bitter melon juice activates cellular energy sensor amp-activated protein kinase causing apoptotic death of human pancreatic carcinoma cells. Carcinogenesis (2013) 34(7):1585–92. doi: 10.1093/carcin/bgt081 PMC3697895 23475945

[16] Hruban RH Gaida MM Thompson E Hong S-M Noe M Brosens LA. Why is pancreatic cancer so deadly? the pathologist’s view. J Pathol (2019) 248(2):131–41. doi: 10.1002/path.5260 30838636

[17] Muhammad N Steele R Isbell TS Philips N Ray RB. Bitter melon extract inhibits breast cancer growth in preclinical model by inducing autophagic cell death. Oncotarget (2017) 8(39):66226–36. doi: 10.18632/oncotarget.19887 PMC5630406 29029506

[18] Wood J Yasmin-Karim S Moreau M Kumar R Akwanwi J Derek A. Characterization of isolated extracts from justicia plant leaves used as remedy for anemia. Molecules (2020) 25(3):534. doi: 10.3390/molecules25030534 31991819 PMC7037932

[19] Munshi A Hobbs M Meyn RE. Clonogenic cell survival assay. In: Chemosensitivity. (2005) Humana Press. p. 021–8. doi: 10.1385/1-59259-869-2:021 15901923

[20] Hill RP. The changing paradigm of tumour response to irradiation. Br inst Radiol (2016) 90:1069. doi: 10.1259/bjr.20160474 PMC5605022 27416998

[21] Mcmahon SJ. The linear quadratic model: Usage, interpretation and challenges. Inst Phys Eng Med (2018) 64(1). doi: 10.1088/1361-6560/aaf26a 30523903

[22] Chadwick KH Leenhouts HP. A molecular theory of cell survival. Inst Phys Eng Med (1973) 18(1):78–87. doi: 10.1088/0031-9155/18/1/007 4803965

[23] Seltzer ES Watters AK Mackenzie D Granat LM Zhang D. Cannabidiol (cbd) as a promising anti-cancer drug. Cancers (basel) (2020) 12(11):3203. doi: 10.3390/cancers12113203 33143283 PMC7693730

[24] Zlotorynski E. The cell cycle flavours of repair. Nat Rev Mol Cell Biol (2016) 17(2):65–5. doi: 10.1038/nrm.2015.24 26695192

[25] Ngwa W Boateng F Kumar R Irvine DJ Formenti S Ngoma T. Smart radiation therapy biomaterials. Int J Radiat Oncol (2017) 97(3):624–37. doi: 10.1016/j.ijrobp.2016.10.034 PMC5302132 28126309

[26] Mueller R Moreau M Yasmin-Karim S. Imaging and characterization of sustained gadolinium nanoparticle release from next generation radiotherapy biomaterial. Nanomaterials (2020) 10(11):2249. doi: 10.3390/nano10112249 33202903 PMC7697013

